# Experimental detection of short regulatory motifs in eukaryotic proteins: tips for good practice as well as for bad

**DOI:** 10.1186/s12964-015-0121-y

**Published:** 2015-11-18

**Authors:** Toby J. Gibson, Holger Dinkel, Kim Van Roey, Francesca Diella

**Affiliations:** Structural and Computational Biology Unit, European Molecular Biology Laboratory, Meyerhofstrasse 1, D69117 Heidelberg, Germany; Health Services Research Unit, Operational Direction Public Health and Surveillance, Scientific Institute of Public Health (WIV-ISP), 1050 Brussels, Belgium

**Keywords:** Linear motifs, Bioinformatics, Molecular switches, Protein complexes, Cell regulation, Experimental design

## Abstract

It has become clear in outline though not yet in detail how cellular regulatory and signalling systems are constructed. The essential machines are protein complexes that effect regulatory decisions by undergoing internal changes of state. Subcomponents of these cellular complexes are assembled into molecular switches. Many of these switches employ one or more short peptide motifs as toggles that can move between one or more sites within the switch system, the simplest being on-off switches. Paradoxically, these motif modules (termed short linear motifs or SLiMs) are both hugely abundant but difficult to research. So despite the many successes in identifying short regulatory protein motifs, it is thought that only the “tip of the iceberg” has been exposed. Experimental and bioinformatic motif discovery remain challenging and error prone. The advice presented in this article is aimed at helping researchers to uncover genuine protein motifs, whilst avoiding the pitfalls that lead to reports of false discovery.

**Electronic supplementary material:**

The online version of this article (doi:10.1186/s12964-015-0121-y) contains supplementary material, which is available to authorized users.

## Background

The molecular deconstruction of cell signalling began in earnest with the identification of regulatory protein kinases and the cloning of the first viral oncogenes, some of which themselves encoded protein kinases captured from cellular signalling systems [1, 2]. During the following decades, a trio of methods-transient overexpression, mutagenesis and western blot-were harnessed together into the main workflow used to investigate regulatory proteins in the cell. In recent years, it has become clear that these methods are inadequate to address the complexity of cell systems, not least because most cellular systems operate under finely balanced gene dosage requirements [3–5] that are obliterated when any one protein is massively overexpressed [6].

A more modern view of cell signalling holds that its elements are highly restricted in space and time [7]. Systematic proteomic studies have forced us to accept that most regulatory proteins spend most of their time in large multi-protein complexes [8–11], increasingly found to be associated with RNA gene products (which we will not address further here) [12]. These complexes are highly dynamic and may coalesce, split apart, relocate, gain and lose individual proteins and, when no longer needed, be fully dismantled. The regulatory decisions emanating from the complexes must then be transmitted to other parts of the cell, for example by detaching a protein from a signalling complex at the plasma membrane and transporting it into the nucleus where it can modulate gene expression, as typified by beta-catenin under Wnt signalling [13].

For the most part, these regulatory complexes are so poorly understood that they are effectively black box input/output devices with little knowledge of the internal workings. Nevertheless, researchers have now provided many examples where small parts of the machinery within subcomplexes have yielded details of information processing mechanisms [14–16]. It turns out that cellular regulatory complexes primarily operate through the assembly and operation of molecular switching mechanisms [17–21]. Therefore, if we desire to fully understand cellular systems, our challenge will be to reveal the full complement of molecular switches specified by the proteome. This number is vast and presently incalculable, but this is our challenge.

**Fig. 1:**
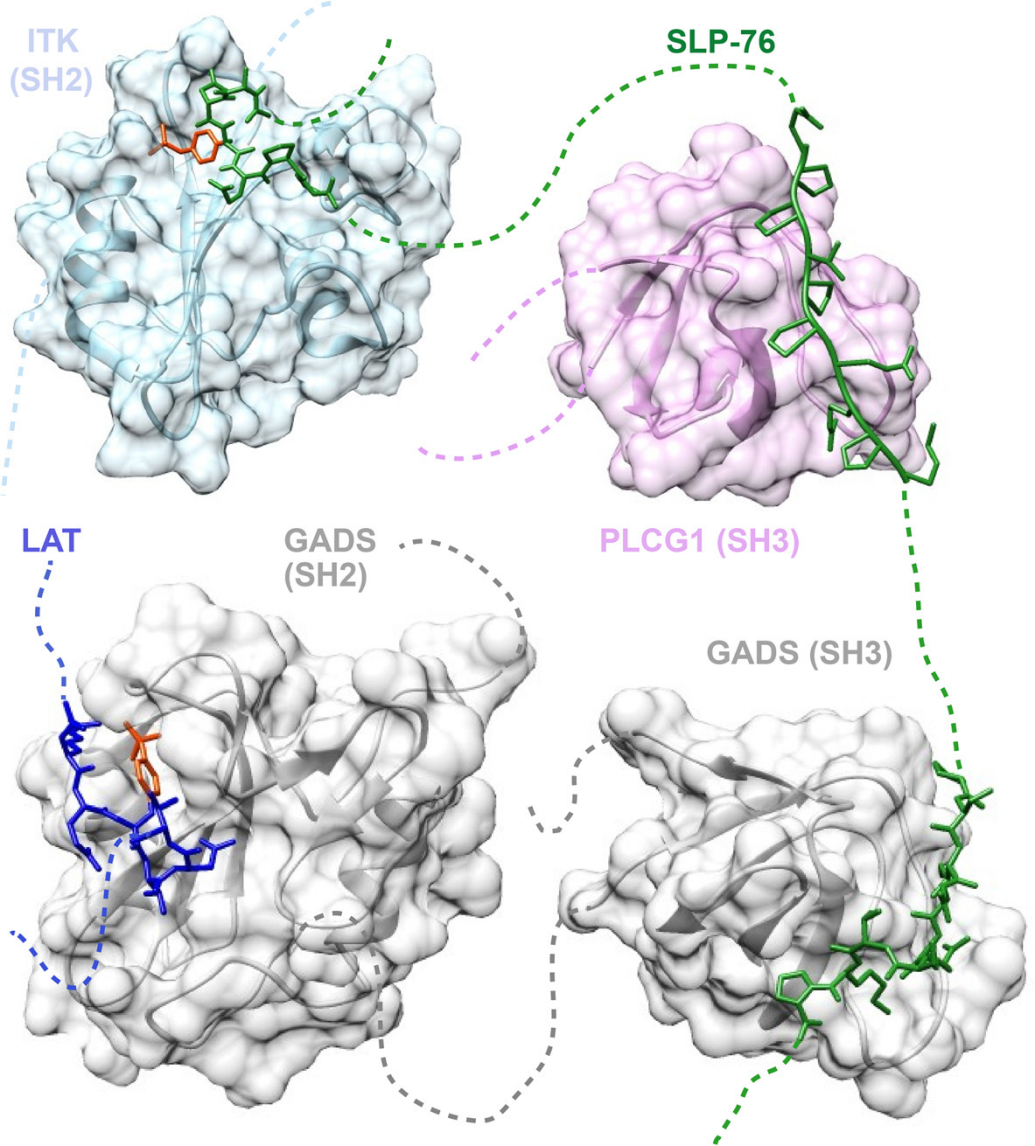
Linear motifs in T cell signalling complex assembly. Four structures of SLiM-domain complexes are combined to show the involvement of motifs in assembly of the T cell receptor signalling complex around the adaptor molecule Linker for activation of T-cells family member 1 (LAT). A phosphorylated SH2 domain-binding motif (YxN) in LAT (189-REYVNV-194, shown in dark blue with the phosphorylated Y191 in red) recruits GRB2-related adapter protein 2 (GADS) via its SH2 domain (grey) (bottom left) (PDB:1R1Q) [79], while the C-terminal SH3 domain of GADS (grey) binds an SH3 domain-binding motif in Lymphocyte cytosolic protein 2 (SLP-76) (233-PSIDRSTKP-241, shown in green) (bottom right) (PDB:2D0N) [80]. Further components are recruited to the complex through other motifs in SLP-76, including an SH3 domain-binding motif (185-QPPVPPQRPM-194, shown in green) that interacts with the SH3 domain of 1-phosphatidylinositol 4,5-bisphosphate phosphodiesterase gamma-1 (PLCG1) (purple) (top right) (PDB:1YWO) [81], and an SH2 domain-binding motif (143-ADYEPP-148, shown in green with the phosphorylated Y145 in red) binding to the SH2 domain of Tyrosine-protein kinase ITK/TSK (ITK) (light blue) (top left) (PDB:2ETZ) [82]

**Fig. 2:**
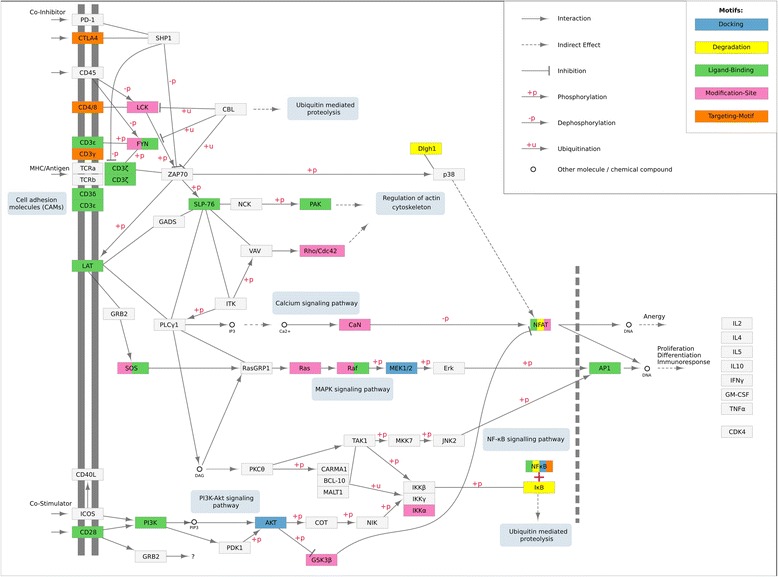
Linear Motifs in T cell receptor signalling pathway hsa04660. “T cell receptor signaling pathway” obtained from KEGG [83] and redrawn using Cytoscape [84] and KEGGScape [85]. Colour coding illustrates the use of linear motifs according to instances annotated in ELM [26] as follows: docking motifs in blue; degradation motifs (degrons) in yellow; ligand-binding motifs in green; sites for post-translational modification in pink; and targeting/trafficking motifs in orange. Note that only motif interactions annotated in the ELM resource have been considered for colouring: Other functionality is not coloured

There appear to be many varieties of molecular switch. Some are regulated by small molecules, for example allosteric switches induced by binding of Ca^++^ ions, GTP or cyclic AMP [22]. Others are effected by cooperative binding interactions of small peptide elements within regulatory protein polypeptide chains. These binding modules are termed short linear motifs or SLiMs. Many regulatory SLiM-mediated interactions are also controlled by one or more of the ~300 different known post-translational modifications (PTMs) [23], further increasing the complexity of switching mechanisms [17, 24]. Figure 1 shows four SLiMs, each in complex with their ligand domain. These interactions assemble a T cell activation complex centred on phosphorylated LAT, a membrane-anchored protein [25]. Many other SLiMs are involved in the T cell signalling network (Fig. 2).

**Fig. 3:**
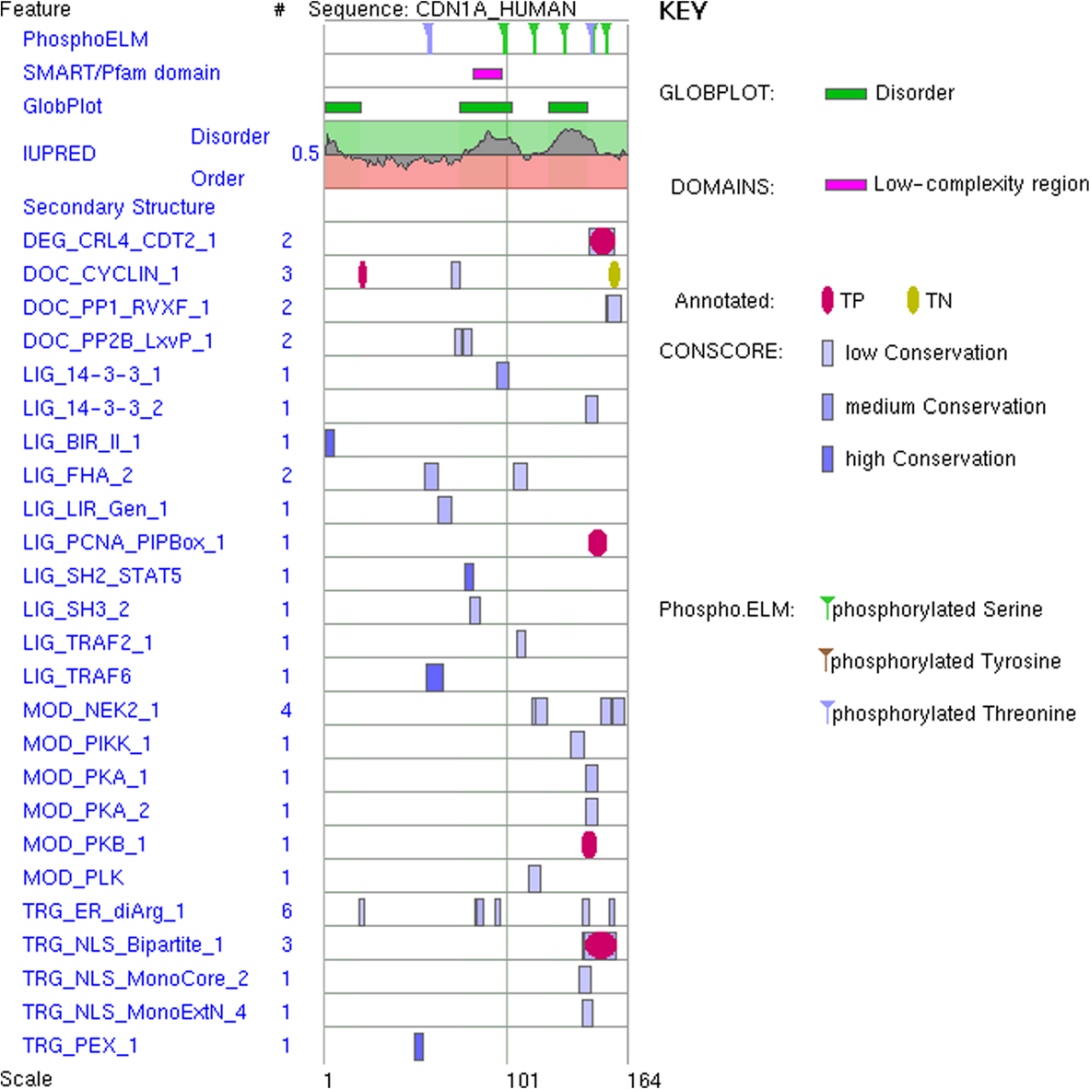
Example of a protein containing multiple linear motifs. Depicted is the output of an ELM [26] query using the p21^Cip1^ Cyclin-dependent kinase inhibitor 1 (Uniprot-Acc:P38936). Upper rows contain annotations/predictions from phospho.ELM [86], SMART [52]/PFAM [51] domain content, and GlobPlot [87]/IUPred [54] disorder predictors. Each subsequent line represents a linear motif class as annotated by ELM with the name on the left side and the instances found depicted on the right side in graphical representation. The already known motifs are annotated (coloured in dark red), the remaining matches (coloured in shades of blue) are candidates of varying likelihood to be real, with one measure being how conserved they are in proteins from other species

Experimentalists teasing apart cell regulatory systems have revealed thousands of examples of these short regulatory protein motifs. Many have been collated into about 250 different pattern variants in the Eukaryotic Linear Motif (ELM) resource, which we provide to the research community [26]. Figure 3 shows ELM output for p21^Cip1^, a small but motif-rich protein that plays a key role in cell cycle checkpoint control. The details of many motif-mediated interactions have been revealed by biochemical, biophysical and structural analyses. But there has also been a prolonged, on-going, persistent and extensive production of false motif literature that confounds attempts to understand regulatory systems [6]. If we can’t prevent this immensely wasteful diversion of scarce resources, perhaps we can at least work to minimise it. Therefore, in this article, we would like to provide guidelines for successful motif discovery and highlight the dangers for the naïve researcher that lead down the path to false discovery.

## Why are there so many SLiMs?

Although there are only around 20,000 protein-coding genes in the human genome, we estimate that the proteome will contain over a million PTM sites plus hundreds of thousands of peptide elements that will become defined as linear motifs [27]. These elements primarily, but not exclusively, reside in segments of intrinsically disordered polypeptide (IDP), i.e., parts of proteins that lack the capability to fold into globular domains. It is estimated that some 30 % of the human proteome cannot adopt a stable, natively folded structure [28, 29]. IDP massively increases the available interaction surface of the proteome with many of those interactions utilising short peptide segments, the linear motifs [30–32]. (In this respect, Eukaryotes are quite different to bacteria, which have limited amounts of intracellular IDP, although there are interesting exceptions such as the degradosome, a very “eukaryotic-like” regulatory complex [33]).

Natural selection acts to optimise organisms to their environment. Over long periods of time, organisms may become increasingly robust to a large variety of environmental parameters. As C. H. Waddington emphasised, natural selection primarily acts to fine-tune weak phenotypes in a process that is both iterative and parallel, such that over time significant phenotypic changes result [34, 35]. As is well understood by engineers, increases in multi-parameter robustness always require increases in system complexity. In the biological context, long-term selection for organismal robustness has been directly responsible for driving an increase in complexity in cell regulatory systems [36]. This has resulted in the modern eukaryotic cell that is full of protein complexes sampling multiple inputs and processing the received information to tune the levels of multiple outputs.

The amount of switching circuitry needed for cellular information processing could not be achieved by complexes consisting solely of globular proteins, which would lack the number of alternative conformational states and alternative interactions needed to control information flow. Instead, it is the IDP elements in regulatory proteins that provide the interaction surfaces enabling system complexity. On their own, however, the flexible IDP elements would confer insufficient precision to the interactions needed to build reliable information processing systems. Therefore, regulatory complexes have an intrinsic duality: structurally precise globular folded domains working with flexible IDPs that enable high information storage, in particular as conditional PTMs [27]. Together they assemble the interconnected dynamic molecular switches that make the regulatory decisions [37].

## If they are so abundant, why are they so hard to find?

A typical short linear motif will have three to four amino acid residues that interact with a part of the surface of the ligand domain [32]. This functionality dictates that these residue positions will be evolutionarily conserved, although some positions may allow a flexible subset of amino acids such as similarly sized hydrophobic side chains (e.g., Ile, Leu, Val) or side chains with similar charge (e.g., Asp, Glu) [38]. A bioinformatician quickly realises that the information content of the sequence space for a given motif (which can be represented by Shannon’s entropy) is remarkably poor and that a proteome will contain such vast numbers of short sequences matching the motif patterns that most cannot be functional. When the number of false positives greatly exceeds the number of true motifs, the poor signal-to-noise ratio will greatly hamper computational discovery of novel motif instances. Consequently, there are still rather few examples of bioinformatic discovery and subsequent experimental validation [39–41]. Similarly, the experimentalist cherry-picking a motif candidate in their favourite protein is also in great danger of going after an invalid target site.

There are at least three reasons why the cell does not get confused by the superabundance of false motif sequences. The first is that signalling is tightly restricted in space and time, such that most false motif-ligand candidates can never physically meet [42]. The second is that many candidate motifs are buried in folded proteins and completely inaccessible to the ligand domain. The third is that even if one false motif were to bind to a partner domain, it will not result in a regulatory event. This is because the typical dissociation constant K_d_ is low micromolar so that the time bound, usually just a few seconds, is far too transient to cause a state change. It is critical to remember that SLiMs always operate cooperatively [8, 20, 32].

## What are the worst mistakes made by experimentalists?

Experimentalists start to go wrong when they overestimate the (normally low) likelihood that any given candidate motif might be real. A lack of understanding of protein sequence/structure relationships and of how sequence evolution and residue conservation can help assessing candidates will mean that the chance to evaluate the protein context will be passed up. There has been a historic tendency to underestimate and even ignore space-time compartmentalisation, naively assuming that a protein with a peptide motif will freely diffuse to find a protein with a partner domain. And there has been a tendency to over-interpret the results of in-cell experiments, which, on their own, can never validate a proposed SLiM-mediated interaction. In past decades, many labs working on signalling protein function used almost exclusively cell cultures and have been unwilling to deploy biochemical, biophysical or structural methodologies. This is unfortunate, as our experience over many years of reviewing the experimental literature for ELM has forced us to conclude that it is essential to undertake in vitro validation of the findings from in-cell work. Given the complexity of macromolecular complexes, a token co-immunoprecipitation using an overexpressed, tagged protein is by no means proof of a motif interaction. While in-cell work is insufficient, so too are purely in vitro binding studies. It is perfectly possible to get an artefactual binding event when combining proteins that never see each other in the cell. For example, actin was first crystallised tightly bound to the secreted bovine gut protein DNAse1 [43].

The key to reliable motif detection is interdisciplinarity: in-cell and in vitro analyses are both needed. If your laboratory is too specialized to handle this, then collaboration with a partner who brings in the complementary expertise is going to be needed.

A key in vitro requirement is to validate the structural integrity of a protein where a candidate motif has been mutated. A significant fraction of SLiMs has two or more conserved hydrophobic residues, for instance, the nuclear export sequence (NES) has four [44]. Most sequence matches to the NES motif are therefore buried in globular protein domains. We have discussed earlier the logical trap where failure to export a mutated protein from the nucleus is taken as proof that a functional NES has been identified [6]. An alternative scenario doesn’t get considered which is that an unfolding mutant of a nuclear protein may accumulate in the nucleus where, if it aggregates, it can no longer leave the compartment. This type of logical error, where a negative result is assumed to provide positive proof of a functional site, can apply to other classes of motif. For example, the D-box anaphase degron has two conserved hydrophobic residues, and thus many candidates are in folded domains. Because amyloids are refractory to proteasomal targeting and destruction [45], persistence of unfolding mutants may be reported as indicative of degron function, when there is no degron at that site [46].

So the worst mistakes made by experimentalists are when they fail to adequately control their experiments by not ensuring that consistent results are obtained from both in vitro and in-cell methods, as well as not checking structural integrity of the mutated proteins.

## Bioinformatics tools that may help motif investigations

In many cases, computational analyses can provide useful guidance as to whether a candidate motif would be worth following up experimentally. There are a number of core activities that should always be done and a much larger number of bioinformatics tools that might sometimes provide extra insight and guidance. We have collected these tools into Table 1, roughly grouped by utility.

**Table 1:** Bioinformatics tools useful for motif discovery. Each resource is listed with its name, weblink, main reference, and short description

Motif Resources/Predictors		
ELM	http://elm.eu.org	[26]
To explore candidate functional sites in proteins and to learn about known motifs		
MiniMotif Miner	http://mnm.engr.uconn.edu	[88]
To analyse protein queries for the presence of short contiguous peptide motifs that have a known function in at least one other protein		
Scansite	http://scansite3.mit.edu	[89]
To identify short protein sequence motifs that are recognized by modular signalling domains, phosphorylated by protein Ser/Thr- or Tyr-kinases or mediate specific interactions with proteins or phospholipids		
PePSite	http://pepsite2.russelllab.org	[90]
To predict binding of a given peptide to a protein structure		
Motif Discovery		
DILIMOT	http://dilimot.russelllab.org	[39]
To find short, over-represented peptide patterns/linear motifs, in a set of proteins		
SLiMFinder	http://bioware.ucd.ie/slimfinder.html	[91]
To find novel, significantly over-represented, short protein motifs		
Sequence Retrieval/Analysis		
BLAST	http://www.uniprot.org/blast http://blast.ncbi.nlm.nih.gov	[47, 92]
To identify regions of local similarity between nuleotide or protein sequences, which can be used to infer functional and evolutionary relationships between sequences as well as help identify members of gene families		
BioMART	http://www.biomart.org	[93]
Provides free software and data services to foster scientific collaboration and facilitate the scientific discovery proces; the project adheres to the open source philosophy that promotes collaboration and code reuse		
Alignment		
Clustal	http://www.clustal.org/omega http://www.ebi.ac.uk/Tools/msa/clustalo	[49, 94]
General purpose DNA or protein multiple sequence alignment program		
MAFFT	http://mafft.cbrc.jp/alignment/server	[95]
Multiple alignment program for amino acid or nucleotide sequences		
Jalview	http://www.jalview.org	[48]
Lightweight Java applet for use in web applications, and a powerful desktop application that employs web services for sequence alignment		
Phylogenetic Tree/Orthology		
TreeFam	http://www.treefam.org	[96]
Database composed of phylogenetic trees inferred from animal genomes, providing orthology/paralogy predictions as well the evolutionary history of genes		
EggNog	http://eggnog.embl.de	[97]
Database of orthologous groups of genes annotated with functional categories derived from COG/KOG categories		
COG	http://www.ncbi.nlm.nih.gov/COG	[98]
Database providing phylogenetic classification of proteins encoded in complete genomes		
Motif Conservation		
Conscore	http://conscore.embl.de	[63]
Linear motif conservation filter		
Consurf	http://consurf.tau.ac.il	[99]
To identify functional regions in proteins		
SLiMPrints	http://bioware.ucd.ie/~compass/biowareweb/Server_pages/slimprints.php	[41]
*De novo* motif discovery tool to identify relatively over-constrained proximal groupings of residues within intrinsically disordered regions, indicative of a putatively functional motif		
Protein Domains		
SMART	http://smart.embl.de	[52]
To identify and annotate genetically mobile domains and to analyse domain architectures		
PFAM	http://pfam.xfam.org	[51]
Database providing a large collection of protein families, each represented by multiple sequence alignments and hidden Markov models		
InterPro	http://www.ebi.ac.uk/interpro	[53]
To classify sequences into protein families and to predict the presence of important domains and sites		
Structure/Disorder		
PDB	http://www.rcsb.org	[55]
Single worldwide repository of information about the 3D structures of large biological molecules, including proteins and nucleic acids		
PDBsum	http://www.ebi.ac.uk/pdbsum	[100]
Pictorial database providing an at-a-glance overview of the contents of each 3D structure deposited in PDB		
IUPred	http://iupred.enzim.hu	[54]
To predict intrinsically unstructured regions in proteins		
D2P2	http://d2p2.pro	[101]
Community resource, providing pre-computed disorder predictions on a large library of proteins from completely-sequenced genomes		
MobiDB	http://mobidb.bio.unipd.it	[102]
Centralized resource for annotations of intrinsic protein disorder		
DISPROT	http://www.disprot.org	[103]
Database providing information about proteins that lack fixed 3D structure in their putatively native states, either in their entirety or in part		
Protein-Protein Interactions		
BioGRID	http://thebiogrid.org	[104]
Online interaction respository with data compiled through comprehensive curation efforts		
STRING	http://string-db.org	[57]
Provides known and predicted protein-protein interactions		
IntAct	http://www.ebi.ac.uk/intact	[105]
Freely available, open source database system and analysis tools for molecular interaction data; all interactions are derived from literature curation or direct user submissions and are freely available		
PiSITE	http://pisite.hgc.jp	[106]
Web-based database of protein interaction sites, providing information on interaction sites of a protein from multiple PDB entries		
DOMINO	http://mint.bio.uniroma2.it/domino	[107]
Database of domain-peptide interactions		
ComPPI	http://ComPPI.LinkGroup.hu	[108]
Cellular compartment-specific database for protein-protein interaction network analysis		
iELM	http://i.elm.eu.org	[109]
Web server to explore short linear motif-mediated interactions		
KEGG	http://www.genome.jp/kegg	[110]
Database resource for understanding high-level functions and utilities of the biological system, such as the cell, the organism and the ecosystem, from molecular-level information, especially large-scale molecular datasets generated by genome sequencing and other high-throughput experimental technologies		
CORUM	http://mips.gsf.de/genre/proj/corum	[56]
Collection of experimentally verified mammalian protein complexes		
Subcellular Localization		
CELLO2GO	http://cello.life.nctu.edu.tw/cello2go	[59]
Web server for protein subcellular localization prediction with functional gene ontology annotation		
LocDB	https://www.rostlab.org/services/locDB	[111]
Database that collects experimental annotations for the subcellular localization of proteins in Homo sapiens and Arabidopsis thaliana		
GeneOntology	http://geneontology.org/ http://www.ebi.ac.uk/QuickGO	[112]
Collaborative effort to address the need for consistent descriptions of gene products across databases		
Compartments	http://compartments.jensenlab.org	[113]
Database of protein subcellular localization data manually curated from the literature or obtained from high-throughput microscopy-based screens		
LOCATE	http://locate.imb.uq.edu.au	[114]
Curated database providing data that describe the membrane organization and subcellular localization of proteins from the RIKEN FANTOM4 mouse and human protein sequence set		
Tissue Expression		
Protein Atlas	http://www.proteinatlas.org	[58]
Publicly available database with millions of high-resolution images showing the spatial distribution of proteins in 44 different normal human tissues and 20 different cancer types, as well as 46 different human cell lines		
TISSUES	http://tissues.jensenlab.org	[115]
Resource integrating evidence on tissue expression from manually curated literature, proteomics and transcriptomics screens, and automatic text mining		
Generic Resources		
UniProt	http://www.uniprot.org	[116]
Manually annotated, non-redundant protein sequence and sequence isoform database; related information about the biological function of protein are curated from the scientific literature		
Antibodypedia	http://www.antibodypedia.com	[117]
Open-access database of publicly available antibodies against human protein targets; contains data on the antibody efficacy in a range of biochemical and cell biological techniques		
IUPAC	http://www.iupac.org	[118]
Serves to advance the worldwide aspects of the chemical sciences and to contribute to the application of chemistry in science		

The key goal is to retrieve as much information as possible about the protein sequence containing the putative motif. A multiple sequence alignment is essential. Sequences can be collected by BLAST-ing [47] with the reference protein. Jalview [48] provides a platform for handling alignments, colour-coding by amino acid similarity and provides web services to remotely interface with alignment software such as Clustal Omega [49] and secondary structure prediction tools such as JPred [50]. Separately, known protein domains can be retrieved from Pfam [51], SMART [52] and InterPro [53]. Native disorder predictors, such as IUPred [54], complement the protein domain and secondary structure predictors. Most (but not all) SLiMs and PTMs are present in IDP. Any site that has been functional over significant evolutionary time periods will show sequence conservation. In fact, it is useful to remember that ALL conserved residues in segments of IDP are functional, whereas many of the conserved residues in globular domains are structural, with primarily those residues at conserved regions of the domain surface being directly functional. The protein structure databank (PDB) [55] should also be checked, as any direct structural knowledge will reinforce (or overrule) the information from the other resources. Protein complex databases like Corum [56] and network/interaction resources such as STRING [57] should be consulted for the known interactors.

Besides the core tools that will always apply for motif discovery, a large number of bioinformatics utilities may optionally come into play (Table 1). For example, if it is not certain whether two proteins are co-expressed in the same cells, the Human Protein Atlas [58] and CELLO2GO [59] might be informative for shared tissue and cellular location. If an antibody is needed for in-cell work, it is worth checking Antibodypedia [60] for user evaluations of antibody quality. Do remember, though, that the information stored in bioinformatics resources is NOT always accurate! Look for synergy between different types of information (as an obvious example, a DNA-binding domain in the protein sequence would synergise with antibody staining that indicated the protein was located in the nuclear compartment). The more critical it is to your project, the more effort you should put into checking up with the primary literature. The next section addresses a specific example of data quality that routinely affects motif discovery.

## Multiple alignments and the choppy state of public sequence data

Most protein sequences in UniProt have been automatically translated from the DNA generated by whole genome sequencing projects using gene prediction algorithms and/or homology to reference sequences. Have you ever wondered how many high quality eukaryotic genome sequences have been produced so far? There are legions of partially finished genomes [61] but the good ones will fit on the fingers of one hand (see also [62]). The way science is set up currently, once the grant has finished, the genome (in whatever state) gets published, usually in a flagship journal, and that is the end of it. There tends to be neither money nor desire to do the unglamorous work needed to finish the job.

It is of course wonderful that we have so much diverse genomic sequence data, allowing research work to be undertaken that was not feasible a few years ago. But the quality issue cannot be avoided and, for most species’ genomes, any gene that is important to your projects should, as a matter of course, be resequenced.

**Fig. 4:**
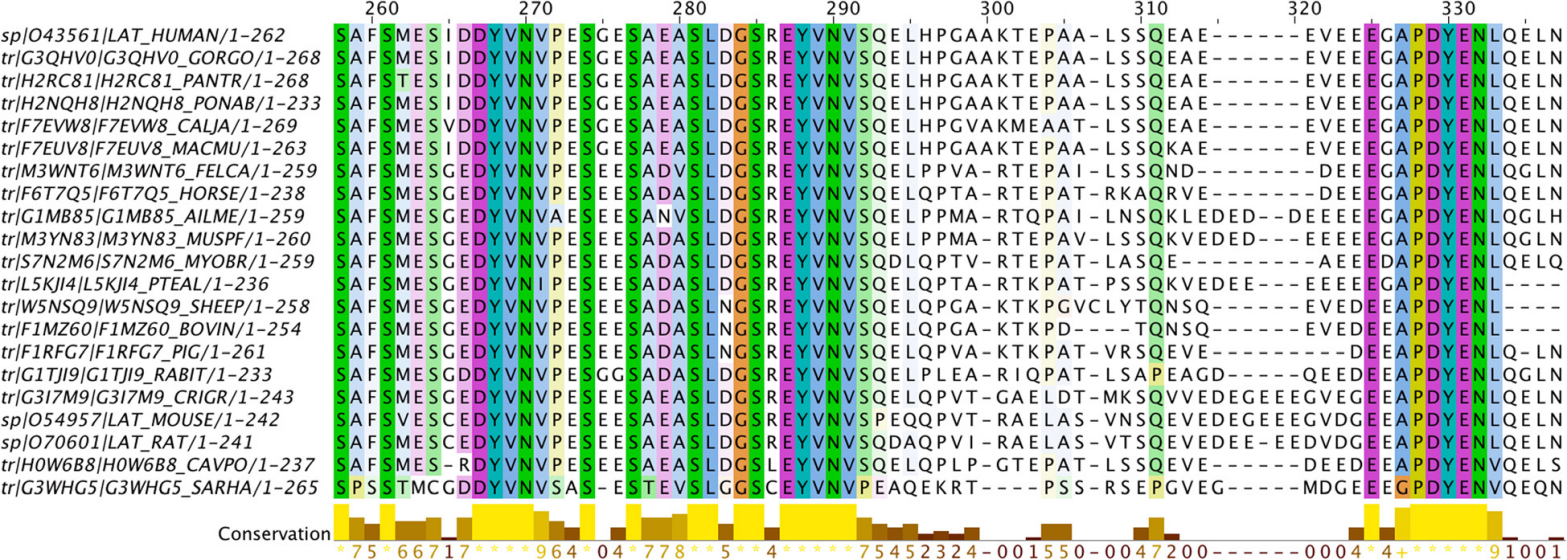
Multiple Sequence Alignment detail for the C-termini of LAT proteins. The three most conserved regions are the critical YxN motifs that bind the GRB2/GADS SH2 domains (see Fig. 1), to assemble the signalling complex. The residue colours are Clustal defaults with less conserved positions faded. LAT protein sequences from representative species were aligned with Clustal Omega [49]. Figure prepared with Jalview [48]

So when we collect a set of available protein sequences and align them, we need to be aware that most of them are low quality and some are very low quality. Base-call errors give the wrong amino acids. N- and C-terminal truncations, missing internal exons abound. Base-dropping/insertion cause frame-shift errors, rendering sections of translated sequence as nonsense. To get a decent quality alignment, you will need to prune out the obviously bad sequences. Make an initial alignment and delete all the silly truncations and missing exons. Now realign the remaining set. Look at the most conserved aligned columns for “impossible” mutations. For example, if zebra has an amino acid not shared by horse and donkey, although the latter are both in agreement with more distantly related mammals, it’s simple: there is probably an error in the zebra sequence, which should not be used in the alignment. Realign your final set of sequences and you are ready to cross-compare your experimental species for conservation of putative motifs. See Fig. 4 for an example alignment of different LAT protein sequences highlighting the important motif residues.

A particular problem for aligning motif-rich sequences is that the alignment programs do not handle natively disordered sequences very well. This is partly because the programs have been optimised to work with globular protein sequences and partly because they expect collinearity of the sequences. An IDP sequence is often more free to tolerate residue substitutions as well as undergo assorted genetic rearrangements. There are likely to be alternatively spliced isoforms, too. Because of these confounding issues, it should not be assumed that the motifs will always be correctly aligned. Even worse, motifs can change position within sequences (probably by duplication and loss of the original) while some motifs are typically found in multiple copies and can vary in number across species [63]. Since motif presence/absence tends to be rather dynamic over long evolutionary timescales, it is generally not useful to align sequences that are too divergent. It should not usually be necessary to drop below ~40 % identity and below ~30 % should be avoided unless there is no choice.

To summarise this section, it is essential to work with multiple sequence alignments. Examine them carefully [64] but at the same time be alert for the many ways that they can also be misleading in the study of motifs.

## Work flows for discovery and validation of short linear motifs

### (a) Developing a work flow for discovery of a new instance of a known motif

Normally the starting point is identification of a candidate motif in a protein of interest. That protein may already be known to interact with the partner protein, or there may be biological plausibility that they might work together, though not yet direct evidence.

**Fig. 5:**
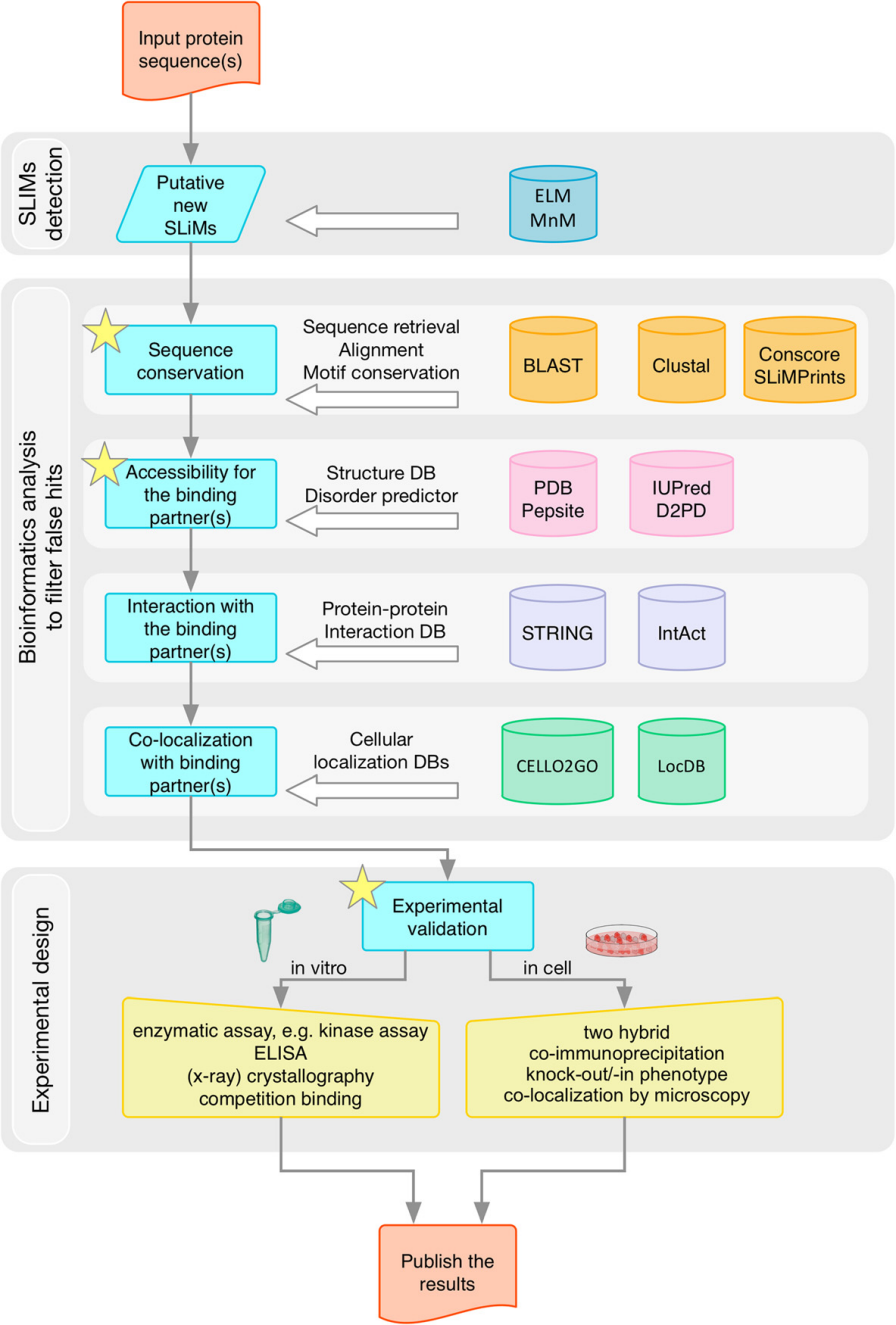
Pipeline for SLiM discovery. Once a candidate sequence location has been identified in a protein, it is evaluated by applying available bioinformatics resources. If the sequence is conserved, accessible to interact and other information is compatible with the motif function, it may pass to experimentation. Both in vitro and in-cell experiments should be undertaken (See Fig. 6 for expanded experimental options). Given a positive outcome of the research it may then be published. On occasion, it may also be of value to publish a negative outcome

**Fig. 6:**
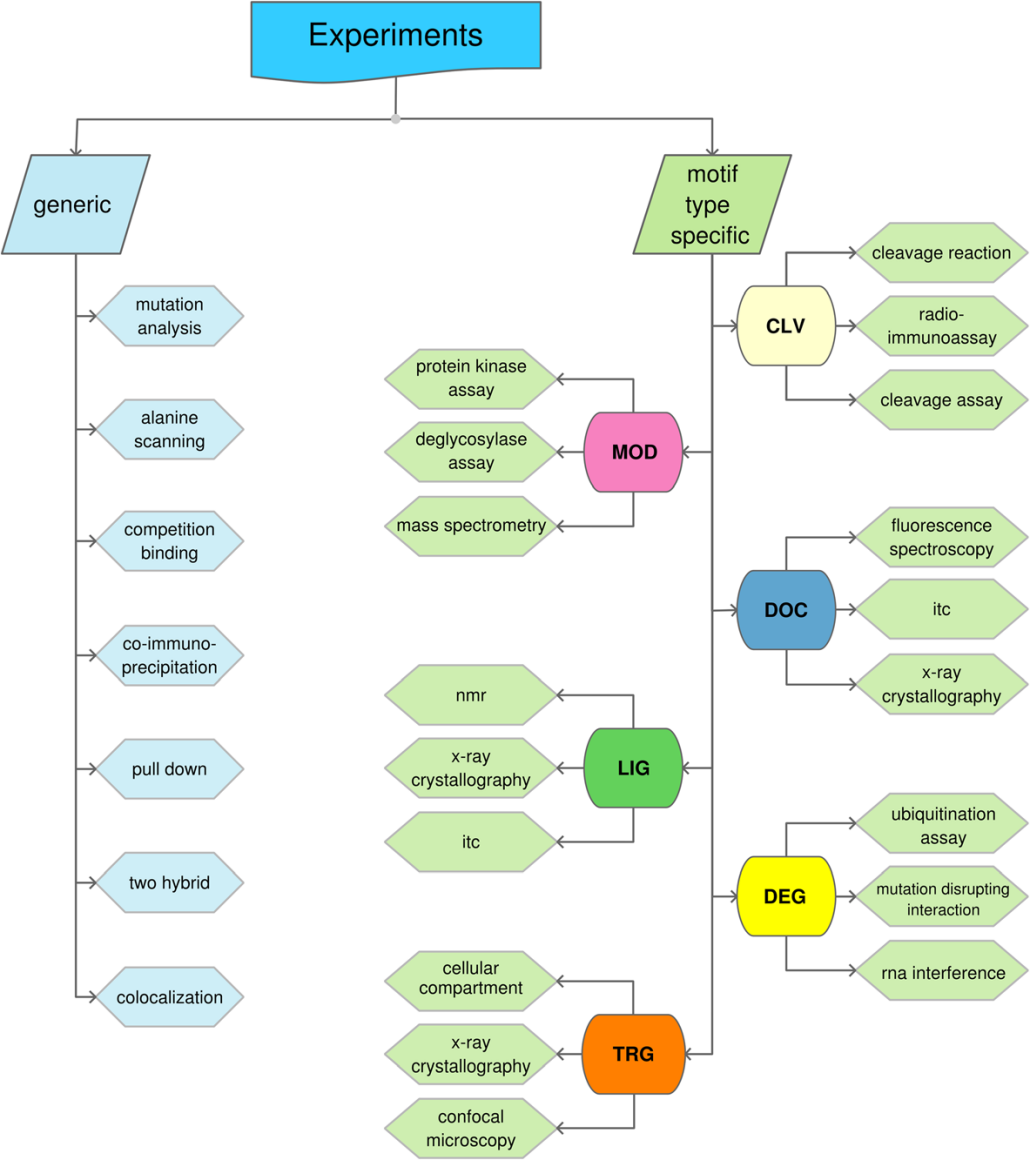
Key experimental approaches to investigate linear motifs. Best-practice experiments to study short linear motifs can be classified into “general” and “motif type-specific”. We highlight a core set of experiments that have been proven to be useful for investigating short linear motif functionality. See the Additional file 1: Table S1 for the list of experiments used in motif discovery, as extracted from the ELM annotation. PSI-MI terms have been used throughout this diagram wherever possible [78]

For a known motif, the residue pattern will usually be well defined, although this is not always the case. Thus, it is worth spending some time confirming the pattern oneself, checking structures and alignments for the key residue positions in the motif. Work through the bioinformatics pipeline indicated in Fig. 5. Not all tools will always apply, but use the ones that do, and perhaps some additional ones from those listed in Table 1, as appropriate. If the motif is buried, or in the wrong cell compartment, or not conserved in related species, these are normally signs to give up now and save time and money. If it is in an alternatively spliced region, this is usually a good sign [65–67]. If all (or most) indications are favourable and you are motivated to do the validation tests, then plan a set of in vitro and in-cell experiments selected from the lists in Fig. 6 and Additional file 1: Table S1 (a list of all experiments that have been annotated in ELM as being in some way relevant to motif discovery). Broadly speaking, there are six functional types of motif [32], and for each of these, specific experiments can be used to validate a functional motif of a given type (Fig. 6), in addition to some more generic experiments that apply to most motifs. It matters for example whether the motif is a targeting signal for subcellular protein localisation, a degron for protein destruction, or a protease cleavage site, so design accordingly. You may wish to purchase peptides for in vitro binding and competition assays and for structural studies. If you can only do a limited set of experiments in your lab, seek a collaborator with complementary expertise. You need to show that there is a relationship between the two proteins being tested, using several different experiments, both in vitro and in-cell. And you need to show that this relationship involves the motif (though of course the interaction doesn’t have to be limited to it, given that these systems are intrinsically cooperative).

### (b) Developing a work flow for de novo motif discovery

Possible starting points for discovering a hitherto unknown variety of protein motif may be a bioinformatics network analysis that places interesting proteins in proximity or, more often, two proteins that are known to directly interact. Subsequently, the two proteins of interest are being chopped up to narrow down the interacting region, guided by the available knowledge of their modular domain architectures, including any solved structures of individual components. If one of those proteins interacts with a region predicted to be within an IDP segment, there may be an embedded linear motif. (If both proteins interact through IDP regions, there may be interacting IDDs - intrinsically disordered domains - as for example in E2F and DP1 and Rb [68].)

Again, performing the bioinformatics analyses (Fig. 5, Table 1) before too much experimentation has been undertaken may be informative for experimental design, as well as saving money and effort if the candidate motif seems implausible. The most conserved region in an interacting IDP segment might include the binding motif.

The experiments are mostly similar to those used to define a new example of an existing motif (Fig. 6, Additional file 1: Table S1). The key difference is the greater uncertainty in the interacting region. As it gets narrowed down, overlapping peptides could be used in binding assays to define the boundaries. Structural studies are extremely desirable, though not always practical in the early rounds of experiments. Nevertheless, there are a number of examples where a solved structure was included in the paper that first defined a novel linear motif [69, 70]. High resolution crystal structures provide the most detailed information of the interaction interface but cannot always be obtained. However, there are also many valuable NMR structures of domain:motif complexes. Again, you need to show that there is a relationship between the two proteins being tested, using several different experiments, both in vitro and in-cell. And you need to show that this relationship involves the motif (though of course the interaction doesn’t have to be limited to a single site, given the cooperative nature of these systems).

If you successfully define a novel linear motif, it is worth using some motif-hunting bioinformatics tools to search for other likely candidates. SLiMSearch for example will rank matches by disorder prediction and conservation [71]. Not all motifs are abundant in the proteome, so there is no guarantee of finding anything. The true motif signal may also be confounded by the noise in the searches. But if you find some candidates, even if you don’t test many or any of them, they will add value when you publish and if others test them, they will increase the citations of your paper.

## Examples of actual linear motif discovery

The ELM resource has over 2400 links to papers either directly detailing SLiM discovery or being relevant to the research area. Thus, researchers can educate themselves on any aspect of experimental motif detection. Still, it might be worth mentioning a couple of high quality examples.

**Fig. 7:**
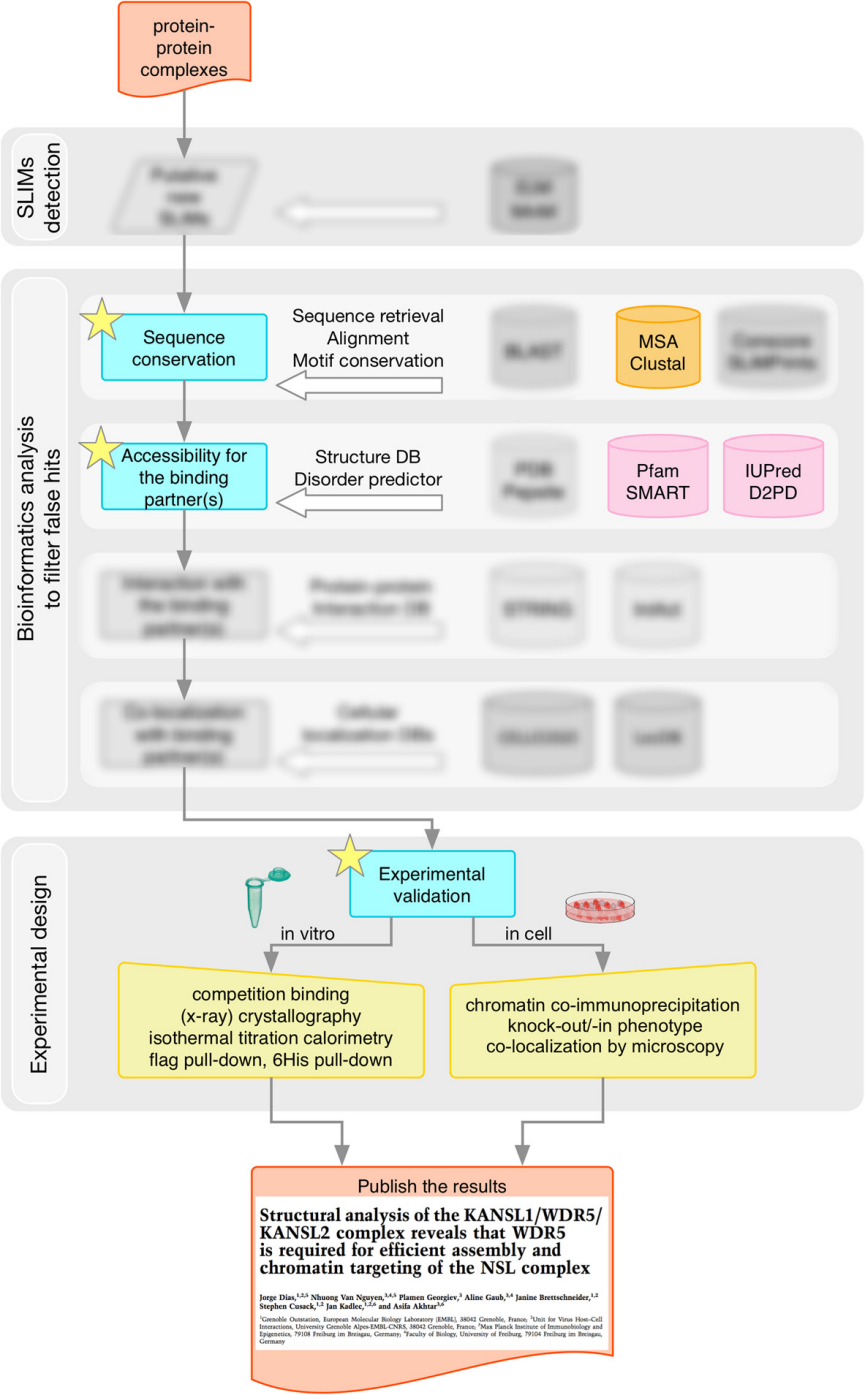
Example of a discovery process mapped onto the pipeline in Fig. 5. Novel motifs were discovered in KANSL1 and KANSL2, binding to different surface locations of the WDR5 protein [69]. Prior knowledge of the NSL protein complex obviated the use of some of the bioinformatics pipeline: these parts are blurred

Novel linear motifs were recently discovered [69] in transcriptional regulatory non-specific lethal complex (NSL) that link the KANSL1 and KANSL2 proteins with WDR5, a protein important in histone modifying complexes. The starting point for defining the interactions was prior knowledge that these proteins interacted as part of NSL function. Testing fragments of the natively disordered parts of KANSL1 provided a ~250 residue interacting region. Mass spectrometry of degraded fragments yielded a minimal binding region. A 14-residue peptide was then successfully crystallised and the structure determined at high resolution. The KANSL2 motif, which binds at a different site on WDR5, was narrowed down by fragment testing, although in this case, the authors may have had some expectation that the motif would be similar to the already known VDV motif of RbBP5. Figure 7 shows the discovery pipeline from above, as implemented in this work, with the unneeded parts of the bioinformatics pipeline blurred out. It should be mentioned that some in-cell work was available from previous publications. Here the in-cell work was taken further, for example including in vivo motif mutation phenotypes in *Drosophila* embryos.

Discovery of the FFAT motif is a good example of a single paper capturing substantial knowledge for a hitherto unknown linear motif [72]. FFAT binds to VAP protein, targeting the motif-containing proteins to the ER membrane. The motif was visualised initially by comparing a 39-residue targeting fragment with a second ER-targeted protein. A range of in-cell experiments using both yeast and mammalian cell systems, such as motif transplantation to GFP and motif mutation, confirmed the motif’s cellular function, targeting to the ER membrane. In vitro binding studies revealed a typical, low-micromolar dissociation constant, while a mutated motif did not bind. A database search using a sequence motif derived from the aligned proteins detected a total of 17 FFAT-containing proteins in vertebrate proteomes with lipid-related functions. Since the motif has six very highly conserved core residues, sequence searches are more informative than for many motifs and so the first paper to discover the motif essentially reported the full set.

We want to conclude this section by noting that methods to show proximity of proteins in-cell are becoming increasingly sophisticated. This means that in future, in-cell proximity might have been “validated” before a motif discovery project is undertaken. In-cell cross-linking Mass Spectrometry is now being performed by a number of labs [73–75]. This technique is undoubtedly challenging but might be indispensible in revealing enzyme-substrate relationships for the ~500 mammalian protein kinases, which fall into large groups with identical or similar target site motifs but very different substrate proteins. Another exciting new method is proximity labelling by biotinylation, BioID [76], which was successfully used recently to identify new substrates targeted to the proteasome for degradation by the betaTrCP E3 ligase [77].

## A rule of thumb 1-2-3 reliability scoring system

As an aide to how well you are doing as well as to judge other motif publications of interest, we provide a simple scoring system for how well-determined a motif is, given the set of experiments undertaken (Table 2). A negative score of minus one would be assigned in case of a violation, for instance for a motif proposed for a protein that comes from the wrong cell compartment, or for one that is well buried within a folded domain. When no evidence is available to support or contradict a candidate motif, a score of zero would be assigned. A score of one would be assigned for minimal and probably indirect evidence in favour of a functional motif, e.g., a co-immunoprecipitation experiment that was abrogated by mutagenesis of the proposed motif or in vitro peptide phosphorylation as the sole evidence. A score of two would indicate that there is good evidence in favour of a functional motif but also some residual uncertainty, for example affinity measurements from peptide binding studies in vitro for proteins that are known to be in the same cell compartment, but without any direct evidence for in vivo function. A maximum score of three, indicating that the motif is beyond reasonable doubt, would require both in-cell and in vitro experimental evidence to show that the purified proteins definitely interact via the motif, that they are certainly co-localized in the cell, that mutation of the motif abrogates function (but remember the caveats discussed above) and, if appropriate, that motif transfer to a new protein brings the function with it.

**Table 2:** Rule of thumb quality scoring scheme

Score	Evidence
−1	Contradictory evidence
0	No evidence
1	Indirect supporting evidence
2	Direct supporting evidence for binding but not for in-cell function
2	Evidence in-cell that proteins associate, but direct supporting evidence for motif binding in vitro is lacking
3	Direct supporting evidence for both binding and in-cell function

## Conclusion

SLiM discovery will continue for many years to be a major activity in research into how cell regulation works. As we have seen, the process has in the past been inefficient and error-prone, so that the literature is full of inadequately characterised motif instances as well as hundreds of false positive identifications. Most of the linear motifs that have been correctly identified so far are in mammalian systems and this bias is reflected in the cellular experimental assays listed. However, yeast and plant researchers will generally have access to equivalent experimental strategies. It is our hope that this article will help researchers to approach motif discovery with good scientific technique, increasing their success rate with the corollary of reducing the wastage of resources that has at times occurred. Their low binding affinities and inherently cooperative nature mean that this is still not necessarily going to be straightforward. But of the million or so motifs used by the cell, the number that are well characterised still just amounts to a rounding error. Good luck hunting them and remember that in science you partly create your own luck according to the quality of the work that you do and the thinking that you put into it.

## Additional file

Additional file 1:
**List of experiments used in SLiM discovery as recorded for experimental instances in the ELM database.** Experimental methods have been grouped by type of motif class (LIG: ligand binding, MOD: modification, TRG: targeting, DOC: docking, DEG: degradation, CLV: cleavage) and sorted by number of instances annotated with this particular method in the ELM database. PSI-MI [78] IDs for experimental methods are given, as are the method classifications. (XLS 59 kb)

## Abbreviations

ELM: Eukaryotic linear motif resource
IDP: Intrinsically disordered polypeptide
PTM: Post-translational modification
SLiM: Short linear motif
NES: Nuclear export signal
SH2: Src Homology 2 domain/motif
SH3: Src Homology 3 domain/motif
CLV: ELM category for cleavage motifs
DEG: ELM category for degradation motifs (degrons)
DOC: ELM category for docking motifs
LIG: ELM category for ligand binding motifs
NSL: Non-specific lethal complex
MOD: ELM category for modification sites
TRG: ELM category for targeting/trafficking motifs

## References

[1] Czernilofsky AP, Levinson AD, Varmus HE, Bishop JM, Tischer E, Goodman HM (1980). Nucleotide sequence of an avian sarcoma virus oncogene (src) and proposed amino acid sequence for gene product. Nature.

[2] de Klein A, van Kessel AG, Grosveld G, Bartram CR, Hagemeijer A, Bootsma D (1982). A cellular oncogene is translocated to the Philadelphia chromosome in chronic myelocytic leukaemia. Nature.

[3] Papp B, Pal C, Hurst LD (2003). Dosage sensitivity and the evolution of gene families in yeast. Nature.

[4] Kaizu K, Moriya H, Kitano H (2010). Fragilities caused by dosage imbalance in regulation of the budding yeast cell cycle. PLoS Genet.

[5] Veitia RA, Potier MC (2015). Gene dosage imbalances: action, reaction, and models. Trends Biochem Sci.

[6] Gibson TJ, Seiler M, Veitia RA (2013). The transience of transient overexpression. Nat Methods.

[7] Scott JD, Pawson T (2009). Cell signaling in space and time: where proteins come together and when they’re apart. Science.

[8] Gibson TJ (2009). Cell regulation: determined to signal discrete cooperation. Trends Biochem Sci.

[9] Breitkreutz A, Choi H, Sharom JR, Boucher L, Neduva V, Larsen B (2010). A global protein kinase and phosphatase interaction network in yeast. Science.

[10] Clancy T, Hovig E (2014). From proteomes to complexomes in the era of systems biology. Proteomics.

[11] Bienz M (2014). Signalosome assembly by domains undergoing dynamic head-to-tail polymerization. Trends Biochem Sci.

[12] Khalil AM, Rinn JL (2011). RNA-protein interactions in human health and disease. Semin Cell Dev Biol.

[13] Clevers H, Nusse R (2012). Wnt/beta-catenin signaling and disease. Cell.

[14] Boja ES, Rodriguez H (2014). Proteogenomic convergence for understanding cancer pathways and networks. Clin Proteomics.

[15] Good MC, Zalatan JG, Lim WA (2011). Scaffold proteins: hubs for controlling the flow of cellular information. Science.

[16] Beck M, Topf M, Frazier Z, Tjong H, Xu M, Zhang S (2011). Exploring the spatial and temporal organization of a cell’s proteome. J Struct Biol.

[17] Van Roey K, Gibson TJ, Davey NE (2012). Motif switches: decision-making in cell regulation. Curr Opin Struct Biol.

[18] Lavoie H, Li JJ, Thevakumaran N, Therrien M, Sicheri F (2014). Dimerization-induced allostery in protein kinase regulation. Trends Biochem Sci.

[19] Chang L, Barford D (2014). Insights into the anaphase-promoting complex: a molecular machine that regulates mitosis. Curr Opin Struct Biol.

[20] Balagopalan L, Coussens NP, Sherman E, Samelson LE, Sommers CL (2010). The LAT story: a tale of cooperativity, coordination, and choreography. Cold Spring Harb Perspect Biol.

[21] Aragon E, Goerner N, Zaromytidou AI, Xi Q, Escobedo A, Massague J (2011). A Smad action turnover switch operated by WW domain readers of a phosphoserine code. Genes Dev.

[22] Motlagh HN, Wrabl JO, Li J, Hilser VJ (2014). The ensemble nature of allostery. Nature.

[23] Choudhary C, Mann M (2010). Decoding signalling networks by mass spectrometry-based proteomics. Nat Rev Mol Cell Biol.

[24] Akiva E, Friedlander G, Itzhaki Z, Margalit H (2012). A dynamic view of domain-motif interactions. PLoS Comput Biol.

[25] Kortum RL, Rouquette-Jazdanian AK, Samelson LE (2013). Ras and extracellular signal-regulated kinase signaling in thymocytes and T cells. Trends Immunol.

[26] Dinkel H, Van Roey K, Michael S, Davey NE, Weatheritt RJ, Born D (2014). The eukaryotic linear motif resource ELM: 10 years and counting. Nucleic Acids Res.

[27] Tompa P, Davey NE, Gibson TJ, Babu MM (2014). A million peptide motifs for the molecular biologist. Mol Cell.

[28] Dunker AK, Lawson JD, Brown CJ, Williams RM, Romero P, Oh JS (2001). Intrinsically disordered protein. J Mol Graph Model.

[29] Ward JJ, Sodhi JS, McGuffin LJ, Buxton BF, Jones DT (2004). Prediction and functional analysis of native disorder in proteins from the three kingdoms of life. J Mol Biol.

[30] Fuxreiter M, Tompa P, Simon I (2007). Local structural disorder imparts plasticity on linear motifs. Bioinformatics.

[31] Pancsa R, Fuxreiter M (2012). Interactions via intrinsically disordered regions: what kind of motifs?. IUBMB Life.

[32] Van Roey K, Uyar B, Weatheritt RJ, Dinkel H, Seiler M, Budd A (2014). Short linear motifs: ubiquitous and functionally diverse protein interaction modules directing cell regulation. Chem Rev.

[33] Bandyra KJ, Bouvier M, Carpousis AJ, Luisi BF (1829). The social fabric of the RNA degradosome. Biochim Biophys Acta.

[34] Waddington CH (1957). The strategy of the genes.

[35] Masel J, Siegal ML (2009). Robustness: mechanisms and consequences. Trends Genet.

[36] Kitano H (2004). Biological robustness. Nat Rev Genet.

[37] Van Roey K, Dinkel H, Weatheritt RJ, Gibson TJ, Davey NE (2013). The switches.ELM resource: a compendium of conditional regulatory interaction interfaces. Sci Signal.

[38] Davey NE, Van Roey K, Weatheritt RJ, Toedt G, Uyar B, Altenberg B (2012). Attributes of short linear motifs. Mol Biosyst.

[39] Neduva V, Russell RB (2006). DILIMOT: discovery of linear motifs in proteins. Nucleic Acids Res.

[40] Di Fiore B, Davey NE, Hagting A, Izawa D, Mansfeld J, Gibson TJ (2015). The ABBA motif binds APC/C activators and is shared by APC/C substrates and regulators. Dev Cell.

[41] Davey NE, Cowan JL, Shields DC, Gibson TJ, Coldwell MJ, Edwards RJ (2012). SLiMPrints: conservation-based discovery of functional motif fingerprints in intrinsically disordered protein regions. Nucleic Acids Res.

[42] McConnachie G, Langeberg LK, Scott JD (2006). AKAP signaling complexes: getting to the heart of the matter. Trends Mol Med.

[43] Suck D, Kabsch W, Mannherz HG (1981). Three-dimensional structure of the complex of skeletal muscle actin and bovine pancreatic DNAse I at 6-A resolution. Proc Natl Acad Sci U S A.

[44] Xu D, Farmer A, Collett G, Grishin NV, Chook YM (2012). Sequence and structural analyses of nuclear export signals in the NESdb database. Mol Biol Cell.

[45] Chakrabarti O, Rane NS, Hegde RS (2011). Cytosolic aggregates perturb the degradation of nontranslocated secretory and membrane proteins. Mol Biol Cell.

[46] Diella F, Haslam N, Chica C, Budd A, Michael S, Brown NP (2008). Understanding eukaryotic linear motifs and their role in cell signaling and regulation. Front Biosci.

[47] Li W, Cowley A, Uludag M, Gur T, McWilliam H, Squizzato S (2015). The EMBL-EBI bioinformatics web and programmatic tools framework. Nucleic Acids Res.

[48] Waterhouse AM, Procter JB, Martin DM, Clamp M, Barton GJ (2009). Jalview Version 2--a multiple sequence alignment editor and analysis workbench. Bioinformatics.

[49] Sievers F, Wilm A, Dineen D, Gibson TJ, Karplus K, Li W (2011). Fast, scalable generation of high-quality protein multiple sequence alignments using Clustal Omega. Mol Syst Biol.

[50] Drozdetskiy A, Cole C, Procter J, Barton GJ (2015). JPred4: a protein secondary structure prediction server. Nucleic Acids Res.

[51] Finn RD, Bateman A, Clements J, Coggill P, Eberhardt RY, Eddy SR (2014). Pfam: the protein families database. Nucleic Acids Res.

[52] Letunic I, Doerks T, Bork P (2015). SMART: recent updates, new developments and status in 2015. Nucleic Acids Res.

[53] Mitchell A, Chang HY, Daugherty L, Fraser M, Hunter S, Lopez R (2015). The InterPro protein families database: the classification resource after 15 years. Nucleic Acids Res.

[54] Dosztanyi Z, Csizmok V, Tompa P, Simon I (2005). IUPred: web server for the prediction of intrinsically unstructured regions of proteins based on estimated energy content. Bioinformatics.

[55] Berman HM, Westbrook J, Feng Z, Gilliland G, Bhat TN, Weissig H (2000). The protein data bank. Nucleic Acids Res.

[56] Ruepp A, Waegele B, Lechner M, Brauner B, Dunger-Kaltenbach I, Fobo G (2010). CORUM: the comprehensive resource of mammalian protein complexes--2009. Nucleic Acids Res.

[57] Franceschini A, Szklarczyk D, Frankild S, Kuhn M, Simonovic M, Roth A (2013). STRING v9.1: protein-protein interaction networks, with increased coverage and integration. Nucleic Acids Res.

[58] Ponten F, Jirstrom K, Uhlen M (2008). The human protein atlas--a tool for pathology. J Pathol.

[59] Yu CS, Cheng CW, Su WC, Chang KC, Huang SW, Hwang JK (2014). CELLO2GO: a web server for protein subCELlular LOcalization prediction with functional gene ontology annotation. PLoS ONE.

[60] Bjorling E, Uhlen M (2008). Antibodypedia, a portal for sharing antibody and antigen validation data. Mol Cell Proteomics.

[61] Genomes Pages - Eukaryota. http://www.ebi.ac.uk/genomes/eukaryota.html. Accessed 4 November 2015.

[62] Nagy A, Patthy L (2013). MisPred: a resource for identification of erroneous protein sequences in public databases. Database.

[63] Chica C, Labarga A, Gould CM, Lopez R, Gibson TJ (2008). A tree-based conservation scoring method for short linear motifs in multiple alignments of protein sequences. BMC Bioinformatics.

[64] Do CB, Katoh K (2008). Protein multiple sequence alignment. Methods Mol Biol.

[65] Weatheritt RJ, Gibson TJ (2012). Linear motifs: lost in (pre)translation. Trends Biochem Sci.

[66] Buljan M, Chalancon G, Eustermann S, Wagner GP, Fuxreiter M, Bateman A (2012). Tissue-specific splicing of disordered segments that embed binding motifs rewires protein interaction networks. Mol Cell.

[67] Ellis JD, Barrios-Rodiles M, Colak R, Irimia M, Kim T, Calarco JA (2012). Tissue-specific alternative splicing remodels protein-protein interaction networks. Mol Cell.

[68] Rubin SM, Gall AL, Zheng N, Pavletich NP (2005). Structure of the Rb C-terminal domain bound to E2F1-DP1: a mechanism for phosphorylation-induced E2F release. Cell.

[69] Dias J, Van Nguyen N, Georgiev P, Gaub A, Brettschneider J, Cusack S (2014). Structural analysis of the KANSL1/WDR5/KANSL2 complex reveals that WDR5 is required for efficient assembly and chromatin targeting of the NSL complex. Genes Dev.

[70] Schuch B, Feigenbutz M, Makino DL, Falk S, Basquin C, Mitchell P (2014). The exosome-binding factors Rrp6 and Rrp47 form a composite surface for recruiting the Mtr4 helicase. EMBO J.

[71] Davey NE, Haslam NJ, Shields DC, Edwards RJ (2011). SLiMSearch 2.0: biological context for short linear motifs in proteins. Nucleic Acids Res.

[72] Loewen CJ, Roy A, Levine TP (2003). A conserved ER targeting motif in three families of lipid binding proteins and in Opi1p binds VAP. EMBO J.

[73] Herzog F, Kahraman A, Boehringer D, Mak R, Bracher A, Walzthoeni T (2012). Structural probing of a protein phosphatase 2A network by chemical cross-linking and mass spectrometry. Science.

[74] Liu F, Rijkers DT, Post H, Heck AJ (2015). Proteome-wide profiling of protein assemblies by cross-linking mass spectrometry. Nat Methods.

[75] Combe CW, Fischer L, Rappsilber J (2015). xiNET: cross-link network maps with residue resolution. Mol Cell Proteomics.

[76] Roux KJ, Kim DI, Raida M, Burke B (2012). A promiscuous biotin ligase fusion protein identifies proximal and interacting proteins in mammalian cells. J Cell Biol.

[77] Coyaud E, Mis M, Laurent EM, Dunham WH, Couzens AL, Robitaille M (2015). BioID-based Identification of Skp Cullin F-box (SCF)beta-TrCP1/2 E3 Ligase Substrates. Mol Cell Proteomics.

[78] Kerrien S, Orchard S, Montecchi-Palazzi L, Aranda B, Quinn AF, Vinod N (2007). Broadening the horizon--level 2.5 of the HUPO-PSI format for molecular interactions. BMC Biol.

[79] Cho S, Velikovsky CA, Swaminathan CP, Houtman JC, Samelson LE, Mariuzza RA (2004). Structural basis for differential recognition of tyrosine-phosphorylated sites in the linker for activation of T cells (LAT) by the adaptor Gads. EMBO J.

[80] Dimasi N (2007). Crystal structure of the C-terminal SH3 domain of the adaptor protein GADS in complex with SLP-76 motif peptide reveals a unique SH3-SH3 interaction. Int J Biochem Cell Biol.

[81] Deng L, Velikovsky CA, Swaminathan CP, Cho S, Mariuzza RA (2005). Structural basis for recognition of the T cell adaptor protein SLP-76 by the SH3 domain of phospholipase Cgamma1. J Mol Biol.

[82] Pletneva EV, Sundd M, Fulton DB, Andreotti AH (2006). Molecular details of Itk activation by prolyl isomerization and phospholigand binding: the NMR structure of the Itk SH2 domain bound to a phosphopeptide. J Mol Biol.

[83] Kanehisa M, Goto S, Sato Y, Kawashima M, Furumichi M, Tanabe M (2014). Data, information, knowledge and principle: back to metabolism in KEGG. Nucleic Acids Res.

[84] Shannon P, Markiel A, Ozier O, Baliga NS, Wang JT, Ramage D (2003). Cytoscape: a software environment for integrated models of biomolecular interaction networks. Genome Res.

[85] Nishida K, Ono K, Kanaya S, Takahashi K (2014). KEGGscape: a Cytoscape app for pathway data integration. F1000Res.

[86] Dinkel H, Chica C, Via A, Gould CM, Jensen LJ, Gibson TJ (2011). Phospho.ELM: a database of phosphorylation sites--update 2011. Nucleic Acids Res.

[87] Linding R, Russell RB, Neduva V, Gibson TJ (2003). GlobPlot: Exploring protein sequences for globularity and disorder. Nucleic Acids Res.

[88] Rajasekaran S, Balla S, Gradie P, Gryk MR, Kadaveru K, Kundeti V (2009). Minimotif miner 2nd release: a database and web system for motif search. Nucleic Acids Res.

[89] Obenauer JC, Cantley LC, Yaffe MB (2003). Scansite 2.0: Proteome-wide prediction of cell signaling interactions using short sequence motifs. Nucleic Acids Res.

[90] Trabuco LG, Lise S, Petsalaki E, Russell RB (2012). PepSite: prediction of peptide-binding sites from protein surfaces. Nucleic Acids Res.

[91] Davey NE, Haslam NJ, Shields DC, Edwards RJ (2010). SLiMFinder: a web server to find novel, significantly over-represented, short protein motifs. Nucleic Acids Res.

[92] Altschul SF, Gish W, Miller W, Myers EW, Lipman DJ (1990). Basic local alignment search tool. J Mol Biol.

[93] Smedley D, Haider S, Durinck S, Pandini L, Provero P, Allen J (2015). The BioMart community portal: an innovative alternative to large, centralized data repositories. Nucleic Acids Res.

[94] McWilliam H, Li W, Uludag M, Squizzato S, Park YM, Buso N (2013). Analysis Tool Web Services from the EMBL-EBI. Nucleic Acids Res.

[95] Katoh K, Standley DM (2014). MAFFT: iterative refinement and additional methods. Methods Mol Biol.

[96] Schreiber F, Patricio M, Muffato M, Pignatelli M, Bateman A (2014). TreeFam v9: a new website, more species and orthology-on-the-fly. Nucleic Acids Res.

[97] Powell S, Forslund K, Szklarczyk D, Trachana K, Roth A, Huerta-Cepas J (2014). eggNOG v4.0: nested orthology inference across 3686 organisms. Nucleic Acids Res.

[98] Tatusov RL, Koonin EV, Lipman DJ (1997). A genomic perspective on protein families. Science.

[99] Ashkenazy H, Erez E, Martz E, Pupko T, Ben-Tal N (2010). ConSurf 2010: calculating evolutionary conservation in sequence and structure of proteins and nucleic acids. Nucleic Acids Res.

[100] Laskowski RA (2001). PDBsum: summaries and analyses of PDB structures. Nucleic Acids Res.

[101] Oates ME, Romero P, Ishida T, Ghalwash M, Mizianty MJ, Xue B (2013). D(2)P(2): database of disordered protein predictions. Nucleic Acids Res.

[102] Potenza E, Di Domenico T, Walsh I, Tosatto SC (2015). MobiDB 2.0: an improved database of intrinsically disordered and mobile proteins. Nucleic Acids Res.

[103] Sickmeier M, Hamilton JA, LeGall T, Vacic V, Cortese MS, Tantos A (2007). DisProt: the Database of Disordered Proteins. Nucleic Acids Res.

[104] Chatr-Aryamontri A, Breitkreutz BJ, Oughtred R, Boucher L, Heinicke S, Chen D (2015). The BioGRID interaction database: 2015 update. Nucleic Acids Res.

[105] Orchard S, Ammari M, Aranda B, Breuza L, Briganti L, Broackes-Carter F (2014). The MIntAct project--IntAct as a common curation platform for 11 molecular interaction databases. Nucleic Acids Res.

[106] Higurashi M, Ishida T, Kinoshita K (2009). PiSite: a database of protein interaction sites using multiple binding states in the PDB. Nucleic Acids Res.

[107] Ceol A, Chatr-aryamontri A, Santonico E, Sacco R, Castagnoli L, Cesareni G (2007). DOMINO: a database of domain-peptide interactions. Nucleic Acids Res.

[108] Veres DV, Gyurko DM, Thaler B, Szalay KZ, Fazekas D, Korcsmaros T (2015). ComPPI: a cellular compartment-specific database for protein-protein interaction network analysis. Nucleic Acids Res.

[109] Weatheritt RJ, Jehl P, Dinkel H, Gibson TJ (2012). iELM--a web server to explore short linear motif-mediated interactions. Nucleic Acids Res.

[110] Tanabe M, Kanehisa M (2012). Using the KEGG database resource. Curr Protoc Bioinformatics.

[111] Rastogi S, Rost B (2011). LocDB: experimental annotations of localization for Homo sapiens and Arabidopsis thaliana. Nucleic Acids Res.

[112] Gene-Ontology-Consortium (2008). The Gene Ontology project in 2008. Nucleic Acids Res.

[113] Binder JX, Pletscher-Frankild S, Tsafou K, Stolte C, O’Donoghue SI, Schneider R (2014). COMPARTMENTS: unification and visualization of protein subcellular localization evidence. Database.

[114] Sprenger J, Lynn Fink J, Karunaratne S, Hanson K, Hamilton NA, Teasdale RD (2008). LOCATE: a mammalian protein subcellular localization database. Nucleic Acids Res.

[115] Santos A, Tsafou K, Stolte C, Pletscher-Frankild S, O’Donoghue SI, Jensen LJ (2015). Comprehensive comparison of large-scale tissue expression datasets. PeerJ.

[116] UniProt-Consortium (2015). UniProt: a hub for protein information. Nucleic Acids Res.

[117] Alm T, von Feilitzen K, Lundberg E, Sivertsson A, Uhlen M (2014). A chromosome-centric analysis of antibodies directed toward the human proteome using Antibodypedia. J Proteome Res.

[118] IUPAC publications. http://www.iupac.org/home/publications.html. Accessed 4 November 2015.

